# Computer‐aided assessment of the chemokine receptors CXCR3, CXCR4 and CXCR7 expression in gallbladder carcinoma

**DOI:** 10.1111/jcmm.15219

**Published:** 2020-06-08

**Authors:** Xiao Yu, Wei Lu, Chan Way Ng, Shuoyu Xu, Yao Wu, Shili Chen, Yuren Gao, Yijian Zhang, Qingqing Zhang, Yuzhen Xu, Yingbin Liu, Sheng Li

**Affiliations:** ^1^ Department of Pathology and Forensic Medicine Dalian Medical University Dalian China; ^2^ Department of General Surgery School of Medicine Xinhua Hospital Shanghai JiaoTong University Shanghai China; ^3^ Institute of Biliary Tract Diseases Research Shanghai JiaoTong University School of Medicine Shanghai China; ^4^ Invitrocue Pte Ltd. Singapore City Singapore; ^5^ Department of Radiology Sun Yat‐sen University Cancer Center Guangzhou China; ^6^ Department of Urology the second Affiliated Hospital of Dalian Medical University Dalian China; ^7^ Department of Gastrointestinal Surgery Xu Zhou Center Hospital Affiliated to Medical College of Southeast University Jiangsu China; ^8^ National-Local Engineering Research Center for Drug Research and Development (R&D) of Neurodegenerative Diesases Department of Biochemistry Dalian Medical University Dalian China

**Keywords:** computer‐aided, CXCR3, CXCR4, CXCR7, gallbladder cancer, quantitative assessment

## Abstract

Gallbladder carcinoma (GBC) is a vicious and invasive disease. The major challenge in the clinical treatment of GBC is the lack of a suitable prognosis method. Chemokine receptors such as CXCR3, CXCR4 and CXCR7 play vital roles in the process of tumour progression and metastasis. Their expression levels and distribution are proven to be indicative of the progression of GBC, but are hard to be decoded by conventional pathological methods, and therefore, not commonly used in the prognosis of GBC. In this study, we developed a computer‐aided image analysis method, which we used to quantitatively measure the expression levels of CXCR3, CXCR4 and CXCR7 in the nuclei and cytoplasm of glandular and interstitial cells from a cohort of 55 GBC patients. We found that CXCR3, CXCR4 and CXCR7 expressions are associated with the clinicopathological variables of GBC. Cytoplasmic CXCR3, nuclear CXCR7 and cytoplasmic CXCR7 were significant predictive factors of histology invasion, whereas cytoplasmic CXCR4 and nuclear CXCR4 were significantly correlated with T and N stage and were associated with the overall survival and disease‐free survival. These results suggest that the quantification and localisation of CXCR3, CXCR4 and CXCR7 expressions in different cell types should be considered using computer‐aided assessment to improve the accuracy of prognosis in GBC.

Gallbladder carcinoma (GBC) is the most prevalent and intractable malignant tumour in the biliary tract, showing a very high metastatic potential and poor prognosis.^1^, ^2^, ^3^ It is important to accurately determine prognostic markers of GBC at an early stage to decide on a more effective treatment plan. Current prognostic markers include chemokines receptors CXCR3, CXCR4 and CXCR7, which are known to be involved in multiple tumour progression and metastasis.^4^ Their tumorigenesis effect is mediated via interactions with the shared ligands ITAC (CXCL11) and SDF‐1 (CXCL12).^5^ It has been reported that CXCR3 is associated with cell proliferation and is involved in the inhibition of cell migration and cancer metastasis in ovarian and prostate cancer cell lines.^6^ CXCR4 was shown to regulate the migration of cancer cells.^7^ CXCR4 expression has been an indicator for the increasing risk of recurrence and poor survival in several malignancies. Similar to CXCR4, CXCR7 plays a role in angiogenesis and tumour growth with its expression correlates with metastasis.^8^ CXCL12 was found to play a crucial role in the progression of GBC by enhancing anchorage‐dependent and anchorage‐independent growth and migration.^9^ Recently, CXCR3, CXCR4 and CXCR7 were shown to have different expressions in the nuclei and cytoplasm.^10^, ^11^, ^12^ Yao et al^13^ conducted a study, in which the expressions of CXCR4 and CXCR7 in gallbladder cancer specimens from 72 patients were analysed, and found that cytoplasmic CXCR4 expression (*P* = .006) and CXCR7 expression (*P* = .035) were independent prognostic factors for survival. However, the conventional assessment of CXCR3, CXCR4 and CXCR7 expression separately does not correlate well with prognosis in GBC.^5^ A more holistic prognostic approach is needed, which this study aims to provide.

The conventional prognostic approach of GBC is using manual immunohistochemistry (IHC). This method has high inter‐ and intra‐observer variations, which makes it not ideal as a standardized indicator for prognosis. Recently, with advances in digital pathology, computer‐aided quantitative image analysis techniques have been adopted for IHC analysis and scoring.^14^, ^15^ These advanced machine learning techniques can detect the nuclei in the whole slide image and quantify staining intensity at single‐cell level after stain deconvolution. Specific tissue regions can be further identified for a more precise assessment of IHC staining.^16^ Observer variation of positive staining is minimized as the cut‐off intensity of the IHC staining is consistent between all the images.

In this study, we investigated the expression and localization of CXCR3, CXCR4 and CXCR7 simultaneously in GBC by tissue microarray (TMA) IHC from a cohort of 55 GBC patients who underwent surgical resection at Xinhua Hospital (Shanghai, China). CXCR3, CXCR4 and CXCR7 were analysed using primary rabbit anti‐human antibody (Sigma‐Aldrich) and secondary antibody‐coated polymer peroxidase complexes (Abcam). To better characterize the histological expression patterns of these molecules with consistent measurements, we developed a quantitative image analysis methodology. This study was approved by the Ethical Committee of Xinhua Hospital affiliated to Shanghai Jiao Tong University School of Medicine. Written informed consent was obtained from all patients. Institutional Review Board approval was obtained for the molecular analysis of tumour blocks.

Both nuclear and cytoplasmic expressions were found for all CXCR3, CXCR4 and CXCR7 analysed (Figure 1). In the regions of glandular cells, CXCR3 and CXCR7 displayed higher nuclear staining (45.3 ± 4.0, 58.2 ± 3.9) than cytoplasmic staining (32.4 ± 3.4, 30.5 ± 3.0) (*P* < .005; Figure 2A,C), while CXCR4 exhibited similar percentage of staining in both nucleus (14.9 ± 2.0, 16.7 ± 2.3) and cytoplasm (*P* = .102; Figure 2B). This finding was similar in the regions of interstitial cells, with a significant higher percentage of positive nuclear staining for CXCR3, CXCR4 and CXCR7 (15.0 ± 2.0, 5.1 ± 1.0, 39.3 ± 4.0) than the cytoplasmic staining (1.6 ± 0.3, 0.9 ± 0.3, 7.6 ± 1.6) (*P* < .005; Figure 2D‐F). Both the nuclear staining and cytoplasmic staining of CXCR3, CXCR4 and CXCR7 were stronger in the glandular cells than in the interstitial cells. The cytoplasmic CXCR3 and CXCR4 staining of interstitial cells was less than 10%. Among CXCR3, CXCR4 and CXCR7, we found that the percentage of positive nuclear and cytoplasmic staining of CXCR4 was lower than the other two chemokines receptors in either glandular cells or interstitial cells.

**FIGURE 1:**
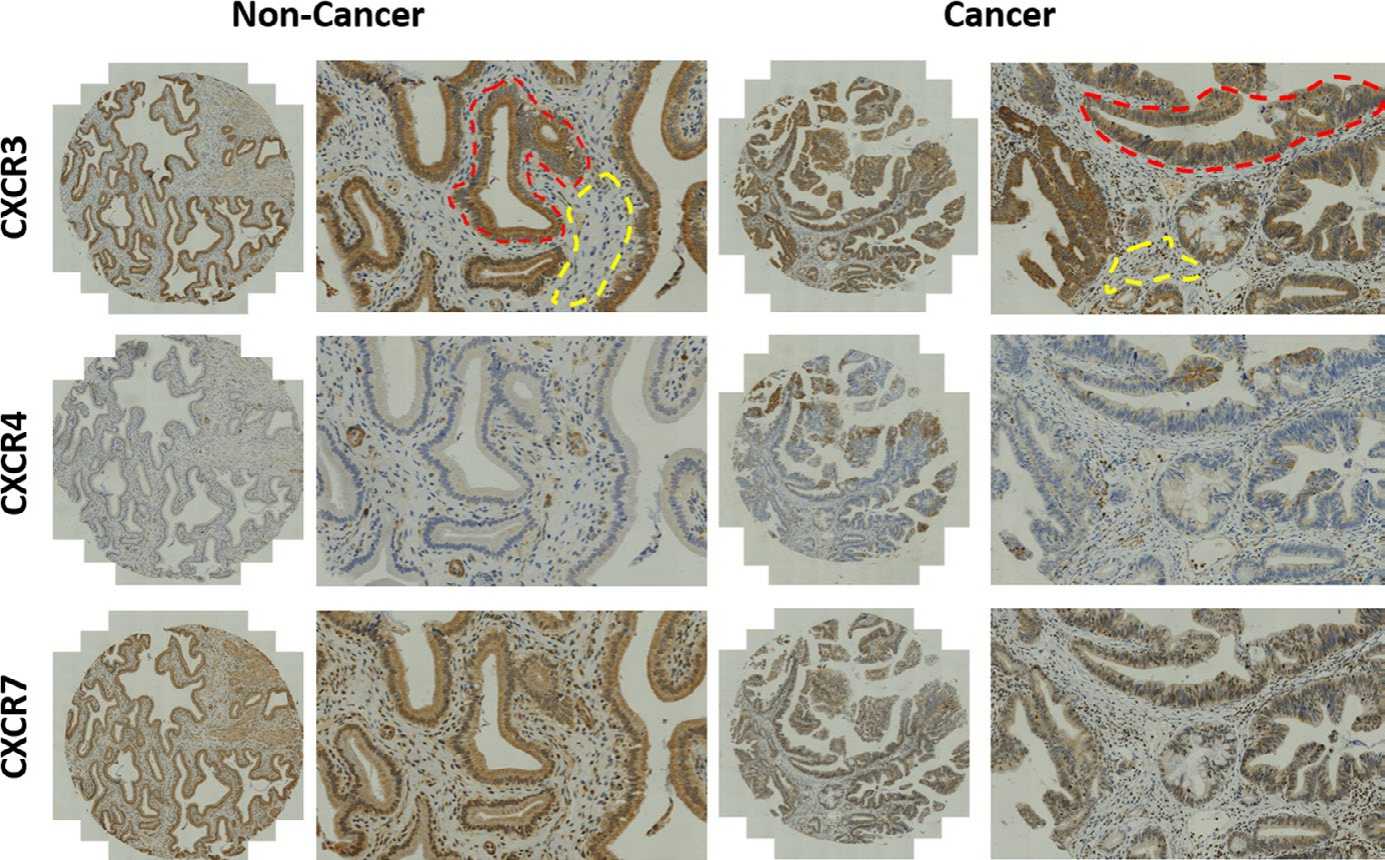
The expression of CXCR3, CXCR4 and CXCR7 in normal and cancerous tissue. The red dotted line outlines a region of interest of glandular cells, and the yellow dotted line outlines a region of interest of interstitial cells

**FIGURE 2:**
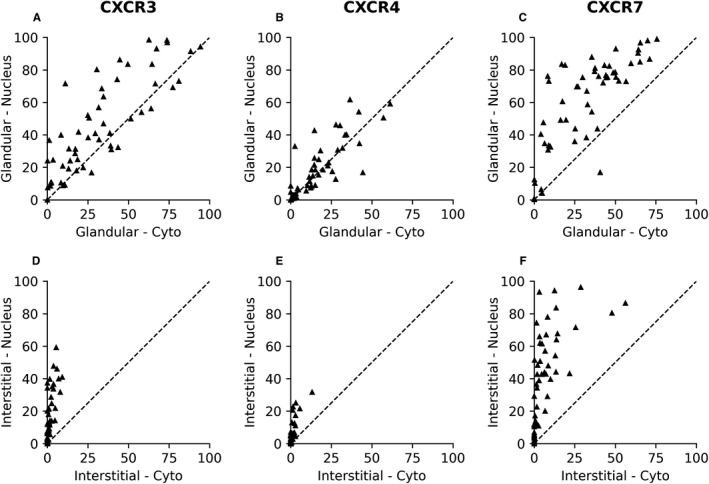
Comparison of positive staining of CXCR3, CXCR4 and CXCR7. (A‐C) percentage of positive staining of CXCR3, CXCR4 and CXCR7 in nuclei and cytoplasm in glandular cell regions; (D‐F) percentage of positive staining of CXCR3, CXCR4 and CXCR7 in nuclei and cytoplasm in interstitial cell regions.

None of the nuclear and cytoplasmic expressions of CXCR3, CXCR4 and CXCR7 in glandular cells and interstitial cells were found significantly different between groups with high and low differentiation score. The logistic regression analysis revealed that none of these measurements were independently correlated with the histology differentiation score.

A similar analysis was performed to correlate expression of CXCR3, CXCR4 and CXCR7 with invasion scores. No significant difference could be observed for the expression of all three chemokines receptors. However, using the logistic regression analysis, we found that the cytoplasmic expression of CXCR3 in glandular cells (*P* = .012) and nuclear and cytoplasmic expression of CXCR7 in glandular cells (*P* = .031 and *P* = .013, respectively) were significant predictors of histology invasion score with AUC around 0.76, indicating a good predictive power.

The samples were categorized into two classes according to the T score, T1‐2 vs. T3‐4. Significant differences were found for the cytoplasmic expression of CXCR3 in glandular cells (*P* = .03) as well as the cytoplasmic and nuclear expression of CXCR3 in interstitial cells (*P* = .03 and *P* = .04, respectively). The results of logistic regression analysis demonstrated that the cytoplasmic and nuclear expressions of CXCR4 in glandular cells (*P* = .013 and *P* = .027, respectively) together with the cytoplasmic expression of CXCR7 in glandular cells (*P* = .042) were independent predictors of the two categories of T score, with the AUC of 0.78.

Next, we categorized the samples according to the N stage, where the first group includes the samples with no regional lymph nodes (N score = 0) and the second group represents the samples with lymph nodes (N score > 0). The cytoplasmic expression of CXCR4 in the glandular cells showed a significant difference between two groups (*P* = .03). Logistic regression analysis consistently showed that the cytoplasmic expression of CXCR4 in the glandular cells was the only independent predictor of N stage (*P* = .021; AUC = 0.688).

The overall TNM score was categorized into early (TNM: 0‐2) and advanced (TNM: 3‐4) stages. Although some of the expression patterns of CXCR3, CXCR4 and CXCR7 were found to correlate with T stage or N stage in the previous study, we did not find significant differences of the expressions of CXCR3, CXCR4 and CXCR7 between early and advanced TNM stage.

The overall survival (OS) and disease‐free survival (DFS) were analysed with nuclear and cytoplasmic CXCR3, CXCR4 and CXCR7 expressions in glandular cells and the clinicopathological variables, including differentiation score, invasion score, T stage, N stage and TNM stage, using the Cox proportional hazards regression analysis. As the sample size was relatively small (N = 55) in this study, we did not include the nuclear and cytoplasmic CXCR3, CXCR4 and CXCR7 expressions in interstitial cells in the analysis to avoid overfitting. Multivariate analysis showed that patient sex, T stage, N stage, the nuclear CXCR7 expression in glandular cells, and the nuclear and cytoplasmic CXCR4 expression in glandular cells were all independently associated with OS. All these six parameters, together with the nuclear CXCR3 expression in glandular cells, were also found to be independently associated with DFS.

In conclusion, we performed a computer‐aided quantitative assessment of the expression of CXCR3, CXCR4 and CXCR7 in GBC that showed that the nuclear and cytoplasmic expressions of CXCR3, CXCR4 and CXCR7 are associated with clinicopathological features and survival, albeit in different ways. The nuclear and cytoplasmic CXCR3 and CXCR7 correlated better with invasion, whereas the nuclear and cytoplasmic CXCR4 were both independently associated to tumour stage and survival. Conventional IHC‐based studies solely analyse one molecular marker, such as CXCR, according to the percentage of positively stained cells to determine its clinical significance. Our computer‐aided quantitative image analysis techniques allow quantitative measurement of the expressions and localization of all three markers together that could improve the prognosis in GBC. Further studies with larger cohorts are required to validate the use of our quantitative assessment of CXCR3, CXCR4 and CXCR7 in GBC prognosis.

## CONFLICTS OF INTEREST

The authors confirm that there are no conflicts of interest.

## AUTHOR CONTRIBUTIONS

Sheng Li and Yinbing Liu contributed to the funding/financial support as well as the conception and design of the study. Wei Lu, Chan Way Ng and Xiao Yu processed the data analysis and interpretation and wrote the manuscript. Shuoyu Xu, Yao Wu, Shili Chen, Yuren Gao, Yijian Zhang, Yuzhen Xu and Qingqing Zhang analysed the correlation between Clustering and Clinical Parameters as well as took part in revision of the manuscript. All authors are responsible for the provision of study materials collection and assembly of data, and final approval of the manuscript.

## Data Availability

The data that support the findings of this study are available on request from the corresponding author. The data are not publicly available due to privacy or ethical restrictions.
